# The Effect of Cadmium on Plants in Terms of the Response of Gene Expression Level and Activity

**DOI:** 10.3390/plants12091848

**Published:** 2023-04-30

**Authors:** Dagmar Moravčíková, Jana Žiarovská

**Affiliations:** Faculty of Agrobiology and Food Resources, Institute of Plant and Environmental Sciences, Slovak University of Agriculture in Nitra, Tr. A. Hlinku 2, 949 76 Nitra, Slovakia; xmoravcikova@uniag.sk

**Keywords:** Cadmium, hyperaccumulators, genes

## Abstract

Cadmium (Cd) is a heavy metal that can cause damage to living organisms at different levels. Even at low concentrations, Cd can be toxic to plants, causing harm at multiple levels. As they are unable to move away from areas contaminated by Cd, plants have developed various defence mechanisms to protect themselves. Hyperaccumulators, which can accumulate and detoxify heavy metals more efficiently, are highly valued by scientists studying plant accumulation and detoxification mechanisms, as they provide a promising source of genes for developing plants suitable for phytoremediation techniques. So far, several genes have been identified as being upregulated when plants are exposed to Cd. These genes include genes encoding transcription factors such as iron-regulated transporter-like protein (ZIP), natural resistance associated macrophage protein (NRAMP) gene family, genes encoding phytochelatin synthases (PCs), superoxide dismutase (SOD) genes, heavy metal ATPase (HMA), cation diffusion facilitator gene family (CDF), Cd resistance gene family (PCR), ATP-binding cassette transporter gene family (ABC), the precursor 1-aminocyclopropane-1-carboxylic acid synthase (ACS) and precursor 1-aminocyclopropane-1-carboxylic acid oxidase (ACO) multigene family are also influenced. Thanks to advances in omics sciences and transcriptome analysis, we are gaining more insights into the genes involved in Cd stress response. Recent studies have also shown that Cd can affect the expression of genes related to antioxidant enzymes, hormonal pathways, and energy metabolism.

## 1. Introduction

Cadmium (Cd) belongs to the group of non-essential elements, which means that plants do not require it for their growth and development. In fact, Cd can have detrimental effects on the normal growth and development of plants [1]. It is harmful to living organisms. In high concentrations it is toxic and can be lethal [2]. Cd is widely distributed in the environment. This toxic heavy metal can be found in soil, air, and water, with the levels influenced by various factors such as natural processes and human activities [3]. As mentioned above, human activity contributes to excessive Cd in soil in addition to natural sources. Careless handling of industrial waste, burning coal, providing nutrients to plants in the form of phosphate fertilisers, and the production of various metals are all associated with ever-increasing levels of Cd in soils [4]. During the previous century, Cd was widely utilised in various industries where Cd-based compounds were prevalent. As we entered the 21st century, more attention was paid to the potential hazards posed by this heavy metal, and materials containing Cd compounds have been phased out if alternative elements that pose no threat to humans, plants, and animals can be employed instead [5]. Nevertheless, even though Cd has been removed from manufacturing processes, research has shown that it still has potential benefits to humans. In particular, recent studies suggest that Cd-containing nanoparticle materials, such as CdO nanoparticles, have exhibited antibacterial properties in the biomedical industry [6]. Cd is also part of today’s so-called fourth generation of solar panels, whose photovoltaic solar cell contains Cd compounds [7]. From an agronomic point of view, the Cd content in soils also negatively affects crop yields [8].

Plants can take up inorganic Cd form from the soil. Cd causes negative morphological as well as physiological changes [8]. Plants differ in their sensitivity to the presence of Cd [9]. Some plants are more resistant while others are more sensitive to elevated levels of Cd in the soil, such as soybeans [10]. Following treatment with Cd, plants typically experience a range of changes, including disruptions to growth parameters, photosynthetic processes, and transport systems in both the phloem and xylem. Additionally, plants may exhibit reduced chlorophyll levels, decreased enzymatic activity, and increased markers of oxidative stress [11]. The changes in root growth are explained in study [12], in which authors came up with an interesting point regarding the amount of Cd in the cells in relation to the phase of cell division. During the phase in which cell division takes place, the S—phase, the number of cells decreased after Cd treatment. This is probably due to the fact that the cell is most sensitive to stressors such as Cd when it is transitioning from initial growth to S—phase.

If a plant is exposed to unfavourable conditions for a long time, it will suffer exhaustion and death [13]. To survive under stress conditions, plants need to respond adequately to the conditions [14]. Plants can also cope with stress more quickly due to their detoxify, chelate and sequester heavy metals [13]. When a plant is exposed to a stressor such as Cd, changes in gene expression occur (Table 1) [14]. By being unable to escape from adverse conditions, plants have to have evolved mechanisms to prevent their demise [15]. Undeniably, Cd affects hormonal genetics pathways and glutathione (GSH) metabolic genes [15,16]. Several transporter gene families whose expression varies in response to Cd exposure are known [17,18,19,20].

The defence mechanisms of plants are still a mystery to us today. Nevertheless, scientists have already uncovered many secrets of the mechanisms of the plant kingdom. However, it is essential to look at these mechanisms at the genetic and molecular levels. This review aims to provide an overview of the genes involved in the stress response following a plant’s exposure to Cd.

## 2. Cd Uptake

**Figure 1:**
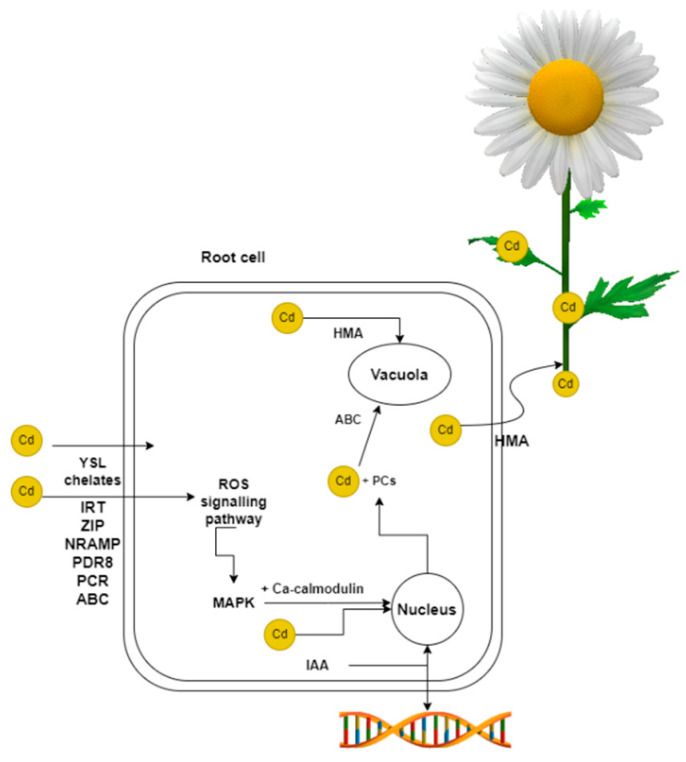
Transcription factors and chelators facilitate the entry of Cd into the plant. Upon Cd entry into the root cell cytosol, the cell activates defense mechanisms. Cd is partly removed by binding to PCs and transported to the vacuole by ABC transporters. HMAs are also involved in the transfer of Cd to vacuoles, as well as to the xylem and subsequently to other parts of the plant. In hyperaccumulators, this transfer to aerial parts takes place. These plants can detoxify or sequester Cd in their leaves. When exposed to Cd, the ROS signalling pathway is activated, involving MAPKs. This pathway, along with Ca-calmodulin, affects the expression of ERFs, MYB, WRKY, and other transcription factor genes. Cd impacts several genes within the cell, although the precise effects are not yet fully comprehended. Plant hormones, such as IAA, also affect gene expression.

The uptake of Cd by plants depends on several factors, including pH. As the soil pH increases, Cd becomes less available to plants and vice versa, the lower the pH, Cd becomes soluble and more readily for plants to take up. Soil type also plays an important role [21], with root secretions released into the soil affecting Cd uptake [22]. The duration of Cd exposure is also important to consider [23,24]. Cd is absorbed by metal transporters in the root of plants (Figure 1). In plants, there are two main pathways by which Cd is transferred, apoplastic and symplastic. These routes differ in terms of how and when the Cd is received and then passed on [25]. When Cd binds to metal transporters, the symplastic pathway is used. It is less demanding for the plant to take up Cd by the apoplast pathway. Cd is taken up by the root surface during H+ exchange after dissociation of carbonic acid [26]. In addition to transcription factors, Cd entry into plant roots is mediated by Cd chelates, in particular by yellow stripe 1-like proteins (YSL) [27]. The conductive network, the xylem, is used for the transfer of Cd from the roots to the stems and leaves [28]. Transfer occurs from root to shoot in hyperaccumulating plants. In the case of non-hyperaccumulating plants, there is no transport of heavy metals from the roots to the aerial parts of the plant [29].

## 3. Hyperaccumulators and Non-Hyperaccumulators

It has long been known that plants have a wide range of mechanisms to protect them against adverse factors. However, the advent of various genomic techniques and omics is helping us to understand these mechanisms at a deeper level, down to the level of regulation of genes involved in the stress response [30].

To this day, numerous plants have been found to possess the ability to hyperaccumulate heavy metals, particularly Cd [14]. Plants capable of accumulating up to 100 mg.kg^−1^ of Cd in their aerial parts are considered to be hyperaccumulators [31]. By utilising genetic tools, it becomes feasible to gain a greater understanding of the mechanisms that plants have evolved to tolerate heavy metals. Of utmost importance is to determine the regulatory mechanisms of the genes involved in the response to heavy metals [14]. In 2015 a new, globally available database was created and named: Global Hyperaccumulator Database (http://hyperaccumulators.smi.uq.edu.au/collection/ (accessed on 3 January 2023)) [32]. Among the representatives of the plant kingdom listed in the database as hyperaccumulators include: *Justicia procumbens* [33], *Bidens pilosa* [34], *Arabis gemmifera* [35], *Noccaea caerulescens*, *Noccaea praecox* [36], *Rorippa globosa* [37], *Sedum plumbizincicola* [38], *Malva sinensis* [39], *Solanum nigrum* [40], *Viola baoshanensis* [41]. However, in fact, many more plants appear to be potential hyperaccumulators. Among the plants that show intriguing results in Cd accumulation belongs *Pinellia ternata* as an intercrop in the crop rotation with *Sedum alfredii* [42].

**Figure 2:**
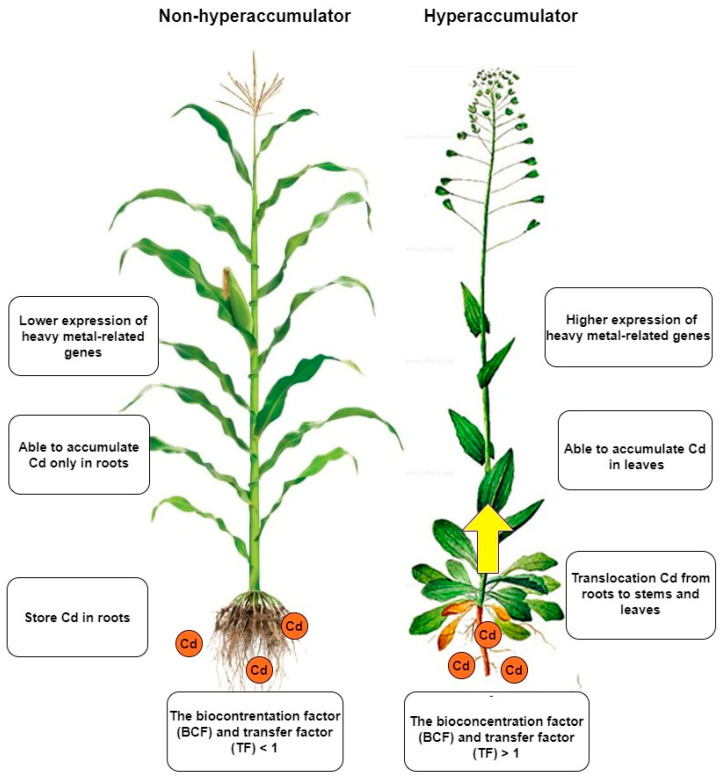
Non-hyperaccumulators and hyperaccumulators differ in several ways. The most significant difference is that hyperaccumulators can transfer Cd to the aerial parts of the plant, allowing them to accumulate Cd not only in the roots but also in the leaves. Additionally, hyperaccumulators can take up much larger amounts of Cd than non-hyperaccumulators. Although both types of plants possess the same genes, the expression of these genes is more tightly regulated in hyperaccumulators when exposed to Cd.

Taking a more detailed look at the disparities between hyperaccumulators and non-hyperaccumulators can unveil the differences in their mechanisms of action (Figure 2). These differences are evident in the way heavy metals are deposited and transferred from roots to aboveground parts, as well as in their antioxidative activity [43]. In contrast, upon comparison of the genetic backgrounds, it can be observed that hyperaccumulator plants possess genes responsible for heavy metal accumulation that are also present in non-hyperaccumulator plants. Nevertheless, the expression and regulation of these genes differ [44,45,46]. These genes are involved in the accumulation, transfer and overall plant detoxification [47]. A comparison between *Arabidopsis thaliana* and *Arabidopsis halleri* showed that the difference between the two plants in their response to heavy metals is likely to be found of gene copies number [48]. Hyperaccumulators also differ in some respect [49]. Differences in tissue-specific expression have been observed in several studies. The reason is that some plants have the ability to move Cd more efficiently from the roots to the aboveground parts [50]. In addition, different amounts of Cd and different lengths of exposure have an effect on the expression [15,16]. The most significant differences can be seen when contrasting two plants with different Cd accumulation capacities [51]. The different amount of Cd in underground parts compared to aboveground parts of the plant is related to Cd transport [12]. Altering of the expression of these transport-related genes not only affects the process of Cd transport and accumulation, but also subsequently disturbs the balance of cations/anions in the cell [52].

Phytoremediation techniques can exploit genes involved in protective mechanisms found in hyperaccumulating plants, not only for Cd but also for other heavy metals [43]. When selecting plants for phytoremediation, it is essential to consider their hyperaccumulative potential. However, it should be remembered that the plant’s applicability is also important and some plants not intended for human consumption may be suitable [22]. For instance, *Malva rotundifolia*, an ornamental plant, was able to accumulate up to 200 mg.kg^−1^ Cd in the underground part and 900 mg.kg^−1^ in the aboveground part during the study, indicating its potential for phytoremediation [53].

## 4. The Effect of Cd on Gene Expression in Plants

Previous studies have shown that Cd negatively affects the regulation of energy metabolism genetics pathways, genetics hormone pathways, enzymatic genetics pathways [54], and phytohormone biosynthesis [54,55]. These ultimately interfere with the expression of genes in response to Cd-induced stress [56]. Furthermore, the glutathione—dependent phytochelatin synthesis pathway is affected. Recent studies no longer target a few stress-related genes but follow the whole transcriptome and provide valuable information about genes related to Cd-induced stress [53]. Various of transporter gene families whose expression varies in response to Cd exposure are known. These gene families include the iron-regulated transporter-like protein (ZIP) gene family, the ATP-binding cassette transporter (ABC) gene family, the cation diffusion facilitator (CDF) gene family and the natural resistance associated macrophage protein (NRAMP) gene family [17,18,19,20,57]. However, also the precursor 1-aminocyclopropane-1-carboxylic acid synthase (ACS) and precursor 1-aminocyclopropane-1-carboxylic acid oxidase (ACO) multigene family [58], genes encoding phytochelatin synthases (PCs) [59] and catalase (CAT) genes are regulated [60]. Even the expression of superoxide dismutase (SOD) genes is affected by Cd [61]. The processes that help plants accumulate and tolerate Cd are receiving a closer look in new research [62]. The simultaneous discovery of several genes involved in the Cd response has been achieved through the study of whole plant transcriptomes. The effect of Cd on genes related to cellulose synthesis has been discovered, including SOD genes, *metallothionein* genes (MT), and genes of myeloblastosis (MYB) transcriptional factor [52]. The formation of secondary metabolites in medicinal plants is also affected at the molecular level after Cd exposure, as evidenced by Artemisia annua L. Upregulation of the following genes was observed: HMGR, FPS, ADS, CYP71AV1, DBR2, ALDH1, DXS and DXR, which are genes involved in artemisinin biosynthesis using quantitative polymerase chain reaction (qPCR) to analyse the upregulation of these genes analysed in the Cd-treated plants [63]. On the one hand, there are variations in the regulation of some genes located in the underground part differing from the regulation of the same genes in the aboveground part of the plant, and on the other hand, some genes show either over or downregulation simultaneously in the stems, leaves and roots, respectively [24,64]. 

New knowledge and conclusions about the harmful effects of Cd on plants have been garnered through various experiments utilising high concentrations of Cd [65]. To gain a comprehensive understanding of the overall pathway in plants following Cd exposure, a more thorough investigation into the mechanisms available to plants in such circumstances is imperative. Two primary systems, enzymatic and non-enzymatic, exist by which plants can mitigate heavy metal stress [66]. The non-enzymatic mechanism means antioxidants such as glutathione and ascorbic acid [67,68]. Reduced GSH, GSH/oxidised glutathione (GSSH) and ratio of total GSH/GSSG is one of the mechanisms involved with a high probability of response after Cd treatment [12,69]. In contrast, the amount of ascorbic acid (ASC) decreased after Cd exposure in many plants such as *Pisum sativum* L., cv Lincoln [69], *Phaseolus vulgaris* L. [70]. Cd caused post-translational modification of CAT [69]. The fact that a plant has multiple mechanisms does not mean that it uses only one to survive [71]. According to a recent study, the cell wall plays an important role in detoxification. It was found that after the plant was exposed to Cd, a large amount of Cd was retained in the cell wall. This means that transport is limited, and the protoplasts are protected [72]. In addition, other mechanisms have been developed to enable the plant to protect itself effectively, such as sequestration in vacuoles, antioxidant mechanism and Cd chelation [73].

Plants encounter a stressor that they must deal with when they are exposed to Cd. Reactive oxygen species (ROS) levels rise in the plant as a result, instigating a chain of reactions. ROS impacts the activity of mitogen-activated protein kinase (MAPK) (Figure 2) [56]. In *Oryza sativa*, two genes encoding *MPKKK17* and *MPK5* are affected by Cd [69]. Triggering of the MAPK cascade is also promoted by plant phytohormones [74]. The reaction continues as MAPK, together with Ca-calmodulin, influences transcription factors notably ethylene response factors (ERFs), MYB, WRKY [54,55,64], the basic helix-loop-helix proteins (bHLH) [64] and isoprenylated plant proteins (HIPPs) [55]. Even brief exposure to Cd has an effect on gene expression. The changes in gene regulation brought about by the indole-3-acetic acid (IAA) pathway are likely the outcome of higher doses of Cd, whereas the changes in regulation resulting from the ROS pathway are triggered by lower Cd doses [75]. The triggering of the cascade and the subsequent influence of transcription factors has on the one hand, a positive effect on the defence mechanism, but on the other hand transcription factors can also cause gene regression. The transcription factor WRKY12 has been identified as one of these negative factors. It is quite likely that the downregulation of genes is caused by being seeded at the W-box in the promoter of a given gene. According to one study, negatively affected genes include *GSH1*, *GSH2*, *PCS1*, and *PCS2* [76]. In *Tritucum durum*, excessive transcription of *TaWRKY74* interferes with the regulating the ascorbate-glutathione (AsA-GSH) cycle genes [77]. Conversely, *Arabidopsis thaliana* shows positive regulation through the transcription factor MYB75, which impacts the expression of the genes ACBP2 and ABCC2 [78]. Hormones also influence gene regulation in *Arabidopsis thaliana* as exemplified by the three metabolic genes GHS: *gsh1*, *gsh2* and *gr1*. Although the expression of these genes has been altered, a study suggests that jasmonic acid (JA) is involved in the plant’s defence mechanism against Cd by regulating GSH genes [16]. Malate dehydrogenase (MMDH2) is engaged in response to Cd exposure. Cd exposure caused downregulation of the *MMDH2*. MMDH2 is part of the Krebs cycle and their downregulation interferes in the next steps of the Krebs cycle. Overall, the study suggests that changes in the regulation of *MMDH2* gene cause differences in the quantity of ROS. This is due to overexpression of the *MMDH2* gene, which affects the conversion of nicotinamide adenine dinucleotide (NADH) to NAD+, when MMDH is abundant, excess NADH is also converted and thus depleted, and there is no NADH left for antioxidant synthesis, which induces excessive stress in plants. However, if *MMDH2* gene expression is repressed, plants become more resistant to Cd. All of this suggests that higher expression of these genes negatively influences plant protection against Cd [59]. In *Arabidopsis thaliana* mutant plants, transcriptomic analysis has been conducted on two genes, GHS1 and GSH2, which are impacted by the transcription factor ZAT6 under Cd-induced stress conditions. ZAT6 binds to the GSH1 gene promoter to initiate the entire process. Additionally, the study suggests that jasmonic acid (JA) is involved in the plant’s defence mechanism against Cd by regulating GSH genes, despite the altered expression of these genes [62].

With the help of transport proteins, a large amount of Cd reaches the vacuoles, where a bond is formed between the metal atom and another molecule with respect to the ion [79]. Cd preferentially binds to PCs and MTs [80]. The role of chelators is also played by HIPPs and HPPs, which are classified as metallochaperones [81]. They form chelates to detoxify the organism [80]. PCs detoxify organisms in such a way that, after binding they are transported by ABC transporters to the vacuole, where cleavage of the Cd-PCs bond occurs, and Cd is stabilised by the ligand-binding pathway. In contrast, MTs detoxify organisms by promoting the production of GSH [82]. HIPPs and HPPs, unlike MTs and PCs, try to remove heavy metals out of the cell in such a way that they transfer these heavy metals to proteins that have an export function and, at the same time, part of the Cd is given to the heavy metal ATPase (HMA) [81]. In plants, many of genes encoding transcription factors have been identified to regulate mechanisms, which plants use to cope with the stress caused by Cd [17].

However, genes are not influenced by just one factor. As an example, changes in the expression level of the *SaHsfA1a*, *SaHsfA2a*, and *SaHsfA4c* genes were observed in *Sedum alfredii* after Cd [83]. It is already known from previous studies that heat-related transcription factors (HSFs) have the capability to influence heavy metals stress-induced genes [77] and there are genes that are also regulated by heat shock. Not only do Cd-treated plants show increased expression but are further enhanced by heat shock [83]. Upregulation of HSFs genes was observed in the root systems of *Oryza sativa* [84] and *Zea mays* [61]. An association between up to eleven heat shock genes and Cd has also been found in *Glycine max*. These findings have implications for the future in the still progressing global warming. Experiments on *Glycine max* described the effect of elevated CO_2_ simultaneously with Cd treatment of plants has a positive effect on the regulation of genes involved in detoxification mechanisms. Such upregulation of genes after the addition of Cd and CO_2_ was also observed for genes encoding NRAMPs, PCs, vacuolar protein sorting-associated protein 2 (VPS2), GSH, glutathione S-transferase (GST), glucose metabolism genes and one gene encoding starch, MAPK, and genes encoding WRKY (*WRKY50*, *WRKY21*, *WRKY58*, *WRKY17*, *WRKY37*, *WRKY51*, *WRKY6*, *WRKY42*, *WRKY25*, *WRKY75*, *WRKY62*) [9].

### 4.1. ATP-Binding Cassette Transporter Gene Family

Plants employ ATP-binding cassette transporters, or ABC transporters, to carry out various functions. One of these functions involves upholding homeostasis when the plant is exposed to heavy metals [57]. The most critical processes that have evolved in plants to protect them from negative effects such as Cd are the *glutathione—dependent* phytochelatin, as has been mentioned and PDR8-dependent pathways [85]. *AtPDR8* is one of the fifteen genes encoding the family of ABC transporters in *Arabidopsis thaliana*, showing overall altered regulation following exposure to external and internal stressors [86]. *WRKY13* has a unique role in response to exposure of Cd. Moreover, their upregulation increases resilience to stress from Cd. They affect upregulation of *PDR8* in *Arabidopsis thaliana*, which decreases accumulation Cd in plants [18]. Plants with overexpression of *MMDH2* have been detected in the regulation of the *PDR8* gene in a negative way [60]. The fact that higher expression of the *PDR8* gene helps plants become more resilient was described. Moreover, it has been concluded that *AtPDR8* acts as a pump for draining excess Cd in *Arabidopsis thaliana* [87].

### 4.2. PCR Gene Family

To date, PCRs have not been sufficiently well studied. However, it has been shown to be able to transfer Cd and zinc (Zn) in plants. They contain the CCXXXXCPC and CLXXXXCPC motifs and also the PLAC8 motif. How much, if at all, these motifs are related to Cd and Zn transfer is not yet known [88]. Several regulated genes from this family of genes have been discovered in a variety of plants. *HvPCR2* plays an important role in the detoxification of *Hordeum vulgare* L. [12]. *SaPCR2* gene is regulated in *Sedum alfredii* [89]. The *OsPCR1* gene has been found to be associated with detoxification and accumulation [90]. Subsequently, another gene was discovered in *Oryza sativa* [51]. The results were obtained using two different plant species, so that the focus was on the difference in their ability to accumulate Cd. Overexpression of the gene *SIPCR6* underpinned the high resistance of *Salix linearistipularis*. Its function was verified using the established transgenic *Populus* [91].

### 4.3. ZIP Gene Family

Homologous genes occurring in the plant kingdom may not show the same expression after exposure to Cd. Evidence shows that expression of homologues ZIP family genes in *Oryza sativa* after Cd treatment is different than in *Arabidopsis thaliana*. The difference is in the location of expression. While in *Arabidopsis thaliana*, there is a higher expression of ZIP genes, especially in the root, in *Oryza sativa*, it is mostly in the shoot. Interestingly, quite the opposite regulation of specific genes: *AtIRT3*, *AtZIP5*, *AtZIP12*, *OsIRT1* and *OsZIP1* was detected [17]. An increase in the expression of the *OsIRT1* and *OsIRT2* genes has been observed in response to Fe deficiency. This overexpression, according to one study, could lead to increased uptake of Cd into the plant. The author goes on to explain that, thanks to OsIRT1, cadmium does not enter the plant through the roots, but is subsequently transported to other parts of the plant as well [92]. *AtZIP9* and *AtIRT1* genes were further detected, where expression was decreased in the roots of *Arabidopsis thaliana*. If a disturbance of expression in *AtFC1* occurs, there is a change in gene transcription.. Detoxification processes in plants with high Cd exposure comprise mainly genes that are involved in GSH-dependent phytochelatin synthetic pathway [93]. A link has been found between *WRKY33* and the expression of *ATL31*, which subsequently affects expression of the *IRT1* gene. The whole mechanism works on the principle of decreasing absorption of Cd. After Cd treatment, gene encodes *WRKY33* is upregulated. In theory, it links to the promotor of the *ATL31* gene. *ATL31* causes degradation of IRT1. Although the IRT1 transporter is the primary carrier for iron, it is also involved in transporting of other metals, including Cd [94]. It is likely that the transfer of a particular metal depends on a conserved residue in or in the immediate vicinity of the transmembrane domain [95]. Cd displaces other elements, such as Fe, Zn and Mn, from the transport pathways [79].

### 4.4. CDF/MTP Gene Family

The CDF gene family is also named MTPs, which means metal tolerance proteins. In *Citrus sinensis* L. overexpression of eight genes was detected in *CitMTP1*, *CitMTP3*, *CitMTP4*, *CitMTP5*, *CitMTP7*, *CitMTP10*, *CitMTP11*, and *CitMTP12* in the underground section of plant. Apart from in the leaves overexpression of five genes was detected in *CitMTP1*, *CitMTP3*, *CitMTP5*, *CitMTP8*, and *CitMTP12*. Samples used in the research was affected by Cd for 7 days and 15 days with the form of Cd 0.038 mM CdSO_4_ and 0.38 mM CdSO_4_. The highest expression was analysed in *CitMTP11* gene in the root with 0.038 mM CdSO_4_ and 15 days affected by Cd [19]. Increases in PCR gene expression were also detected in the sensitive plant *Glycine max*. In the leaves were upregulated genes *GmaMTP1.1*, *GmaMTP1.2*, *GmaMTP3.1*, *GmaMTP3.2* and *GmaMTP4*, whereas in root were upregulated *GmaMTP1.1*, *GmaMTP1.2*, *GmaMTP3.1*, *GmaMTP4.1*, *GmaMTP4.3*, *GmaMTP10.4*, and *GmaMTP11.1* [10]. In Fagopyrum tartaricum one gene *FtMTP8.2* has been detected so far [96]. In *Medicago truncatula* there are twelve known CDS genes, of which five respond to Cd. *MtMTP1.2* and *MtMTP4* are upregulated in the root, *MtMTP1.2* and *MtMTP4* in stems, *MtMTP4* in leaves. Based on bioinformatics analyses, it grouped all of the MTP genes in *Medicago truncatula* into clusters, and within these clusters, the genes showed a high degree of concordance. Further analyses showed that these genes contain domains that can be influenced by abiotic stressors and hormones [97].

### 4.5. NRAMP Gene Family

Many NRAMP genes have been identified in plants so far. These NRAMP genes are famous for encoding metal transporters. The study of plant genomes using modern techniques has led to new insights into the adaptive processes that have played an important role during evolution [98]. Phylogenetic studies show that the number of introns and motif in the NRAMP genes family is changing. This information could mean that plants are adapting to stresses during evolution. A consequence of NRAMP genes being able to respond to heavy metals-induced stress is that the promoters of these genes contain many motifs or elements. In a comparative study, the MYB MYC and STRE elements were the most abundant in the observed *Spirodela polyrhiza* plant. In addition, ABRE motifs linked with the hormones were present in the compared plants. Following Cd exposure, the expression of NRAMP genes in the plant is altered. The genes *SpNramp1*, *SpNramp2*, and *SpNramp3* of Spirodela polyrhiza are sensitive to Cd treatment. These genes showed their activity mainly in the root [99]. In *Arabidopsis thaliana*, NRAMP genes AtNRAMP2, AtNRAMP3, AtNRAMP4 and AtNRAMP5 contain up to 67–75% conserved regions in amino acid sequence. After Cd treatment overexpression, the *AtNRAMP3* gene causes changes in Fe accumulations and root growth [20]. That this gene is involved in the maintenance of metal homeostasis is also demonstrated by Oomen [100]. Furthermore, the function of these genes in response to heavy metals was analysed in *Thlaspi caerulescens*. Expression of NRAMP3 and NRAMP4 was significantly higher in *Thlaspi caerulescens* than in *Arabidopsis thaliana* [100]. It has been discovered that there is a difference in gene expression, specifically *NRAMP1*, *NRAMP2*, *NRAMP3*, *NRAMP5* and *NRAMP6* in two varieties of peppers [72]. *OsNRAMP1* has also been discovered in Oryza sativa which is associated with increased uptake and accumulation in the plant [101].

### 4.6. ACS and ACO Multigene Family

It is established that ACO and ACS share a common role in the production of ethylene [102] and that the overexpression of their genes by Cd leads to an increase in the production of ethylene. The further determined genes which are involved in response to heavy metals have been demonstrated in previous studies. An increase of gene expression of *ACS1*, *ACS2*, *ACS4*, *ACS5*, *ACS6*, *ACS7*, *ACS8* was identified in *Arabidopsis thaliana* after Cd treatment. While the higher level of the expression in the root was *ACS6* after 72 h, in the leaves was after 24 h. These studies have also observed increased expression in members of the ACO multigene family. The most highly expressed genes, *ACO2* and *ACO4* were detected in the roots and in leaves after Cd treatment [58].

### 4.7. HIPP/HPP Gene Family

The number of genes encoding HIPP/HPP in plants is very high. In some plant species, there are more than two hundred [103]. The *OsHIPP56* gene is thought to play an important role. For testing this, the CRISPR/Cas9 technique was used to create rice in which this gene was knocked out. The mutation resulted in large amounts of Cd entering the edible parts of the plant [104].

### 4.8. PCs Gene Family

The *PCS1* and *PCS2* genes have also been implicated in the response to Cd in plants defence mechanisms. However, the transcriptomic level is also influenced by ZAT6, which belongs to the ZIP transporter family [62]. Strikingly, in the study by Santoro [64] in the plant *Arundo donax* L., Cd did not alter the expression of PCS genes. This is a consequence of the fact that in this experiment, the plant defence mechanism was not triggered by PCS [64]. Following the application of 50 μM Cd in *Vicia sativa*, the *VsPC1* gene was upregulated, probably leading to increased tolerance to Cd [105]. It is suggested that *PCS1* is affected by overexpression of the *MAN3* gene. *MAN3* in *Arabidopsis thaliana* is important in responding to heavy metal stress. After exposure to Cd, there is an increase in the expression of *MAN3*. *MAN3* has a critical role in increasing mannose concentration and the consequent activation of PC synthesis related genes such as *PCS1* and *PCS2* [106]. This whole cascade likely contains up to four different components involved in responding to Cd. This entire complex includes the MYB4 transcription factor, which binds to the *MAN3* gene and the MAN3-mediated mannose-binding-lectin 1 (MNB1) gene. It is the MNB1-related GNA domain that influences the resistance of this complex, as it is capable of association with MAN3 [107].

### 4.9. MT Gene Family

MTs provide sites on their genes for transcription factors to bind, resulting in differential expression and response to heavy metal exposure. A differential expression of the *ZmMTs* has been found in *Zea mays*. In the underground part of the plant, the expression of *ZmMT3* and *ZmMT7* genes is reduced. On the one hand, a higher expression of *ZmMT3*, and lower expression of *ZmMT1*, *ZmMT7* and *ZmMT8* were observed in the stems. In the leaves, a different expression was observed. The *ZmMT3*, *ZmMT7* and *ZmMT9* genes were more highly expressed and the *ZmMT1* and *ZmMT8* genes were less highly expressed [108]. Additional studies identified the *Spirodela polyrhiza SpMT2a* gene, which is highly likely to be involved with Cd tolerance based on expression at the transcriptome level after 24 h [109]. The genes *MTB2*, *MTB3* and *MTB15* were found to be upregulated in *Calotropis gigantea* L. [24]. In *Oryza sativa* and *Triticum aestivum*, there is a relatively high probability that *HsfA4a* influences the MT genes, and thus enhances the defence of the plants [110].

### 4.10. Antioxidant Genes

The presence of SOD genes in plants enhances their ability to withstand stress caused by different abiotic factors [111]. The presence or expression of antioxidant enzymes such as SOD can enhance the plant’s ability to resist damage caused by Cd exposure. However, the expression of genes related to antioxidant enzymes such as POD, CAT, APX, FeSOD, and MnSOD varies with the concentration of Cd present. Interestingly, these genes do not exhibit similar expression patterns and respond differently to varying levels of Cd. These observations have been validated in the case of *Lolium perenne* L. [112]. Following Cd treatment in *Lolium perenne* L., overexpression of SOD genes was observed. These genes encode different isoforms of SOD, including Cyt Cu/ZnSOD, MnSOD, and ChlCu/ZnSOD, each with unique expression patterns. The maximum expression of *Cyt Cu/Zn SOD* gene is between 6–24 h, while the maximum expression of *MnSOD* is between 4–6 h and the overexpression of *MnSOD* is also between 4–6 h [113]. In another plant, many SOD genes have been studied that are essential for the response to Cd treatment. *Kandelia obovata* was also examined for the expression of a family of SOD genes. A recent study has provided insight into the *KoCSD1*, *KoCSD2*, *KoCSD3*, *KoFSD1*, *KoFSD2*, *KoFSD3* and *KoMSD* genes and their expression after exposure to Cd. By means of the quantitative RT-PCR method, they conclude that each of these genes is upregulated after Cd treatment. There was a significant upregulation in the roots and in the above-ground parts of *Kandelia obovata*. There has been an interesting breakthrough about gene expression in medical plants [61]. Two years later, selected *KoFSD2* and *KoCSD3* genes were published and transferred into *Nicotiana bethamiana*, where they came up with the idea that it was *KoCSD3* that might have a function in plant defence, preventing Cd from reaching the roots by affecting the roots at the cellular level. Its overexpression in the root exodermis confirmed this discovery [114]. Gene *BjCAT3* from *Brassica juncea* was studied in connection with Cd exposure. The changes of expression were observed by the Northern block. To confirm the association of this gene with Cd, a transgenic plant was generated where higher expression of this gene was shown to be associated with Cd [60].

### 4.11. HMA Gene Family

HMA genes, particularly *HMA1* and *HMA2*, were overexpressed in another study [72]. Several genes in plants such as *NcHMA4* and *NcHMA3* in *Noccaea caerulescens*, *AhHMA3 Arabidopsis halleri* in shoots [49], *SpHMA7* [115] and *SpHMA3* in *Sedum plumbizincicola* [38], *OsHMA3* in *Oryza sativa* [116] were reported. A detailed analysis of all eight HMA-encoding genes was performed for *Sedum plumbizincicola*. This led to the conclusion that these genes contain the elements DKTGT, GDGxNDxP, PxxK S/TGE, HP and CPx/SPC [115]. Twenty-one genes encoding HMA have been identified in *Hordeum vulgare* L., Hv. From this number, five HMA genes were selected that had changes in regulation by Cd [117]. Analyses comparing HMA-encoding genes in *Arabidopsis thaliana* and *Brassica rapa* var. *rapa* showed that the genes in *Brassica rapa* var. *rapa* had undergone evolutionary changes. The genes have been separated into two groups. Different heavy metals species are associated with each group. Out of a total of fourteen genes, upregulation changes were observed for four genes *BrrHMA1*, *BrrHMA2.1*, *BrrHMA2.2* and *BrrHMA4.1*, which were observed in roots of Cd-resistant plants. The upregulation of the *BrrHMA2.2* gene in the root was observed in the Cd-sensitive variety, while over expression of the other genes was not observed here. One gene, *BrrHMA1*, showed the opposite expression in the sensitive plant, where it was downregulated, and conversely upregulated in the resistant plant [118]. Table 1 is a summary of the genes that were affected by Cd treatment. The genes are divided into groups called families. These are shown in the first column. The second column is the plant in which the gene was under regulation and the third column is the gene identification number from the NCBI database [119]. The fourth column gives the name of the gene and the fifth column gives the gene’s function in relation to Cd treatment.

## 5. Conclusions and Future Perspective

It should be emphasised that a plant’s capacity to withstand heavy metal exposure relies on the expression of genes responsible for regulatory pathways, as well as for synthesising metabolites and proteins that actively respond to stress induced by heavy metals [125]. Using transgenic plants, we have revealed that the overexpression of genes involved in the response following Cd exposure prevents the uptake of Cd into the plant [126,127]. There are certain genes that have demonstrated promising outcomes in transgenic plants, such as *TdSHN1*, a transcription factor gene from *Triticum durum*. However, it is important to take a comprehensive approach and consider all the mechanisms involved in detoxification, accumulation, and other rescue mechanisms, rather than just focusing on individual genes in isolation. It would also be pertinent to investigate the impact of DNA methylation on defence mechanisms, as studies have shown that it plays a role in the plant defence system [128]. Cis-analyses have led to the conclusion that genes that are affected by abiotic stress contain cis-acting elements, which would explain their functions [115]. A more comprehensive knowledge of the genetic and molecular processes involved in plants may aid us in developing genetically engineered plants that possess genes capable of meeting the phytoremediation standards. These genes play a vital role in inhibiting Cd toxicity by participating in the accumulation, transport, and detoxification processes in plants. Singh [43] point out that some plants are only tolerant to certain metals, and the problem arises when several undesirable metals are present in the soil at the same time. However, it is important to keep in mind that everything has limits. The study by Wojas [50] where the overexpression of genes that protect plants against Cd toxicity has a positive effect on a plant on the one hand, but on the other hand, the transfer of its gene and the creation of a transgenic plant does not always have a positive effect.

Acquiring a thorough understanding of how plants respond to abiotic stresses is crucial. The expansion of industrialisation and urbanisation has led to a rise in heavy metal pollution, particularly Cd contamination in soil. Addressing these issues is critical to prevent Cd from entering the food chain, but it necessitates a significant amount of knowledge. Although there is still a long way to go, it is necessary to continue our efforts to acquire this knowledge. Plants face toxicity from Cd even at low concentrations, but they have developed several mechanisms to deal with it. When Cd enters the plant through the roots, it can be transported to the aerial parts of the plant, but not all plants can achieve this. The ability to do so is influenced by the expression of genes that play a role in the relevant processes. It is crucial to investigate genes that are involved in the uptake, translocation, and stabilisation of heavy metals in plants to understand how plants maintain homeostasis at the molecular-genetic level to prevent damage. Studying these genes can provide valuable insights into their roles in different plants, which can be used in phytoremediation techniques in combination with genetic engineering to clean up contaminated soils and maintain overall soil health, ultimately preventing the entry of pollutants into the food chain. Future research should aim to examine the plant as a whole, taking into account all aspects, rather than focusing on individual levels and parts in isolation.

## Figures and Tables

**Table 1:** Genes regulated by Cd treatment.

Gene Family	Plant	Gene ID	Gene	Function	Reference
**ABC**	*Arabidopsis thaliana*	BK001007.1	*AtPDR8*	Cd transport	[87]
	*Oryza sativa*	4327728	*ABCG36*	Cd transport	[57]
**ACS**	*Arabidopsis thaliana*	825324	*ACS1*	Cd tolerance	[58]
	*Arabidopsis thaliana*	837082	*ACS2*	Cd tolerance	[58]
	*Arabidopsis thaliana*	816812	*ACS4*	Cd tolerance	[58]
	*Arabidopsis thaliana*	836709	*ACS5*	Cd tolerance	[58]
	*Arabidopsis thaliana*	826730	*ACS6*	Cd tolerance	[58]
	*Arabidopsis thaliana*	828726	*ACS7*	Cd tolerance	[58]
	*Arabidopsis thaliana*	829933	*ACS8*	Cd tolerance	[58]
**Antioxidant genes**	*Lolium perenne L.*	N/A	*MnSOD*	Cd tolerance	[112]
	*Kandelia obovata*	N/A	*KoFSD2*	Cd tolerance	[114]
	*Kandelia obovata*	N/A	*KoCSD3*	Cd tolerance	[114]
**CDF/MTP**	*Citrus sinensis* L.	N/A	*CitMTP1*	Cd tolerance	[19]
	*Citrus sinensis* L.	N/A	*CitMTP3*	Cd tolerance	[19]
	*Citrus sinensis* L.	N/A	*CitMTP4*	Cd tolerance	[19]
	*Citrus sinensis* L.	N/A	*CitMTP5*	Cd tolerance	[19]
	*Citrus sinensis* L.	N/A	*CitMTP7*	Cd tolerance	[19]
	*Citrus sinensis* L.	N/A	*CitMTP10*	Cd tolerance	[19]
	*Citrus sinensis* L.	N/A	*CitMTP11*	Cd tolerance	[19]
	*Citrus sinensis* L.	N/A	*CitMTP12*	Cd tolerance	[19]
	*Citrus sinensis* L.	N/A	*CitMTP8*	Cd tolerance	[19]
	*Glycine max*	N/A	*GmaMTP1.1*	Cd tolerance	[10]
	*Glycine max*	N/A	*GmaMTP1.2*	Cd tolerance	[10]
	*Glycine max*	N/A	*GmaMTP3.1*	Cd tolerance	[10]
	*Glycine max*	N/A	*GmaMTP3.2*	Cd tolerance	[10]
	*Glycine max*	N/A	*GmaMTP4*	Cd tolerance	[10]
	*Glycine max*	N/A	*GmaMTP4.3*	Cd tolerance	[10]
	*Glycine max*	N/A	*GmaMTP10.4*	Cd tolerance	[10]
	*Glycine max*	N/A	*GmaMTP11.1*	Cd tolerance	[10]
	*Fagopyrum tartaricum*	N/A	*FtMTP8.2*	Cd tolerance	[96]
	*Medicago truncatula*	N/A	*MtMTP1.2*	Cd tolerance	[97]
	*Medicago truncatula*	N/A	*MtMTP4*	Cd tolerance	[97]
	*Medicago truncatula*	N/A	*MtMTP1.2*	Cd tolerance	[97]
	*Medicago truncatula*	N/A	*MtMTP4*	Cd tolerance	[97]
**HMA**	*Oryza sativa*	4342783	*OsHMA3*	Cd translocation, acccumulation	[120]
**MT**	*Sedum plumbizincicola*	MK893990.1	*SpMT2*	Cd detoxification	[121]
	*Zea mays*	N/A	*ZmMT3*	Cd tolerance	[108]
	*Zea mays*	N/A	*ZmMT7*	Cd tolerance	[108]
	*Zea mays*	N/A	*ZmMT1*	Cd tolerance	[108]
	*Zea mays*	N/A	*ZmMT7*	Cd tolerance	[108]
	*Zea mays*	N/A	*ZmMT8*	Cd tolerance	[108]
	*Spirodela polyrhiza*	N/A	*SpMT2a*	Cd tolerance	[109]
	*Calotropis gigantea*	N/A	*MTB2*	Cd tolerance	[24]
	*Calotropis gigantea*	N/A	*MTB3*	Cd tolerance	[24]
	*Calotropis gigantea*	N/A	*MTB15*	Cd tolerance	[24]
**NRAMP**	*Arabidopsis thaliana*	841127	*AtNRAMP2*	Cd transport	[20]
	*Arabidopsis thaliana*	816847	*AtNRAMP3*	Cd transport	[20]
	*Arabidopsis thaliana*	836868	*AtNRAMP4*	Cd transport	[20]
	*Arabidopsis thaliana*	827613	*AtNRAMP5*	Cd transport	[20]
	*Arabidopsis thaliana*	838166	*AtNRAMP6*	Cd transport	[122]
	*Glycine max*	100812381	*NRAMP2A*	Cd transport	[23]
	*Glycine max*	100815628	*NRAMP5A*	Cd transport	[23]
	*Glycine max*	100789871	*NRAMP1B*	Cd transport	[23]
	*Glycine max*	100791117	*NRAMP3A*	Cd transport	[23]
	*Glycine max*	100797298	*NRAMP6A*	Cd transport	[23]
	*Oryza sativa*	4342862	*OsNRAMP1*	Cd transport	[101]
	*Spirodela polyrhiza*	N/A	*SpNramp1*	Cd transport	[99]
	*Spirodela polyrhiza*	N/A	*SpNramp2*	Cd transport	[99]
	*Spirodela polyrhiza*	N/A	*SpNramp3*	Cd transport	[99]
	*Capsicum annuum*	N/A	*NRAMP1*	Cd transport	[72]
	*Capsicum annuum*	N/A	*NRAMP2*	Cd transport	[72]
	*Capsicum annuum*	N/A	*NRAMP3*	Cd transport	[72]
	*Capsicum annuum*	N/A	*NRAMP5*	Cd transport	[72]
	*Capsicum annuum*	N/A	*NRAMP6*	Cd transport	[72]
**PCR**	*Oryza sativa*	N/A	*OsPCR1*	Cd detoxification	[90]
	*Salix linearistipulari*	N/A	*SlPCR6*	Cd detoxification	[91]
	*Hordeum vulgare* L.	N/A	*HvPCR2*	Cd detoxification	[12]
	*Sedum alfredii*	N/A	*SaPCR2*	Cd detoxification	[89]
**PCs**	*Arabidopsis thaliana*	831845	*PCS1*	Cd detoxification	[62]
	*Arabidopsis thaliana*	839354	*PCS2*	Cd detoxification	[62,123]
	*Arabidopsis thaliana*	828409	*GSH1*	Cd tolerance	[76]
	*Arabidopsis thaliana*	832797	*GSH2*	Cd tolerance	[76]
	*Arabidopsis thaliana*	842387	*AtIRT3*	Cd transport	[17]
**ZIP**	*Arabidopsis thaliana*	836336	*AtZIP12*	Cd transport	[17]
	*Arabidopsis thaliana*	N/A	*AtZIP5*	Cd transport	[17]
	*Arabidopsis thaliana*	829439	*AtZIP9*	Cd transport	[93]
	*Arabidopsis thaliana*	827713	*AtIRT1*	Cd transport	[93]
	*Arabidopsis thaliana*	820457	*AtZIP1*	Cd transport	[93]
	*Oryza sativa*	4333669	*OsIRT1*	Cd transport	[92,124]
	*Oryza sativa*	4333667	*OsIRT2*	Cd transport	[92]
	*Oryza sativa*	AY324148.1	*OsZIP1*	Cd transport	[17]
**Energetic pathway**	*Oryza sativa*	4342404	*LOC4342404*	Unknown	[54]
	*Oryza sativa*	4347395	*LOC4347395*	Unknown	[54]
	*Oryza sativa*	4334300	*LOC4334300*	Unknown	[54]
	*Oryza sativa*	4352085	*LOC4352085*	Unknown	[54]
	*Oryza sativa*	4335799	*LOC4335799*	Unknown	[54]
	*Oryza sativa*	4342192	*OS07G0105600*	Unknown	[54]
	*Oryza sativa*	4329766	*LOC4329766*	Unknown	[54]
	*Oryza sativa*	4344281	*LOC4344281*	Unknown	[54]
	*Oryza sativa*	4347336	*LOC4347336*	Unknown	[54]
	*Oryza sativa*	4344281	*LOC4344281*	Unknown	[54]
**Signalling pathway**	*Oryza sativa*	4347336	*LOC4347336*	Unknown	[54]
	*Oryza sativa*	4324556	*LOC4324556*	Unknown	[54]
	*Oryza sativa*	4332175	*OS03G0235000*	Unknown	[54]
	*Oryza sativa*	4332175	*LOC4332175*	Unknown	[54]
**Peroxidase pathway**	*Oryza sativa*	4337483	*LOC4337483*	Unknown	[54]
	*Oryza sativa*	4337892	*LOC4337892*	Unknown	[54]
	*Oryza sativa*	4350051	*LOC4350051*	Unknown	[54]
	*Oryza sativa*	4349585	*LOC4349585*	Unknown	[54]
	*Oryza sativa*	4324556	*LOC4324556*	Unknown	[54]
	*Oryza sativa*	4332175	*OS03G0235000*	Unknown	[54]
	*Oryza sativa*	4332175	*LOC4332175*	Unknown	[54]
	*Oryza sativa*	4337483	*LOC4337483*	Unknown	[54]
	*Oryza sativa*	4337892	*LOC4337892*	Unknown	[54]
	*Oryza sativa*	4350051	*LOC4350051*	Unknown	[54]
	*Oryza sativa*	4349585	*LOC4349585*	Unknown	[54]

## Abbreviations

ACO	precursor 1-aminocyclopropane-1-carboxylic acid oxidase
ACS	precursor 1-aminocyclopropane-1-carboxylic acid synthase
APX	ascorbate peroxidase
ASC	ascorbic acid
bHLH	basic helix-loop-helix
bZIP	basic leucine zipper
CAT	catalase
Cd	cadmium
CDF/MTS	metal tolerance protein
ERFs	ethylene response factors
ERP	ethylene-responsive factor
GSH	glutathione
GSSG	oxidised glutathione
GST	glutathione S-transferase
HIPPs/HPP	heavy metal-associated isoprenylated plant proteins
HMA	heavy metal ATPase
IAA	indole-3-acetic acid
JA	jasmonic acid
MAPK	mitogen-activated protein kinase
MTP	metal tolerance protein
MTs	metalothionein
MYB	myeloblastosis
NRAMP	Natural resistance associated macrophage protein
PCR	cadmium resistance family
PCs	phytochelatin synthases
POD	class III peroxidase
ROS	reactive oxygen species
SOD	superoxide dismutase
ZIP	iron-regulated transporter-like protein
